# Tetra­kis[μ-4-(dimethyl­amino)benzoato-κ^2^ *O*:*O*′]bis­[(*N*,*N*-diethyl­nicotinamide-κ*N* ^1^)zinc(II)]

**DOI:** 10.1107/S1600536809047473

**Published:** 2009-11-14

**Authors:** Tuncer Hökelek, Hakan Dal, Barış Tercan, Özgür Aybirdi, Hacali Necefoğlu

**Affiliations:** a Department of Physics, Hacettepe University, 06800 Beytepe, Ankara, Turkey; b Department of Chemistry, Faculty of Science, Anadolu University, 26470 Yenibağlar, Eskişehir, Turkey; c Department of Physics, Karabük University, 78050 Karabük, Turkey; d Department of Chemistry, Kafkas University, 63100 Kars, Turkey

## Abstract

The title mol­ecule, [Zn_2_(C_9_H_10_NO_2_)_4_(C_10_H_14_N_2_O)_2_], is a centrosymmetric binuclear complex, with Zn atoms [Zn⋯Zn′ = 2.8927 (4) Å] bridged by four carboxyl­ate groups from the dimethyl­amino­benzoate (DMAB) ligands. The four carboxyl O atoms around the Zn atom form a distorted square-planar arrangement; the distorted square-pyramidal coordination geometry is completed by the pyridine N atom of the *N*,*N*-diethyl­nicotinamide (DENA) ligand. The Zn atom is displaced by 0.3326 (2) Å from the plane of the four O atoms, with an average Zn—O distance of 2.0416 (12) Å. The dihedral angles between the carboxyl­ate groups and the adjacent benzene rings are 5.31 (8) and 11.00 (9)°, while the pyridine ring is oriented at dihedral angles of 66.26 (6) and 37.88 (7)° with respect to the benzene rings. Weak intra­molecular C—H⋯O and inter­molecular C—H⋯π inter­actions are present.

## Related literature

For general background to niacin and the nicotinic acid derivative *N*,*N*-diethyl­nicotinamide (DENA), see: Bigoli *et al.* (1972 ▶); Krishnamachari (1974 ▶). For related structures, see: Hökelek *et al.* (1995 ▶, 2009*a* ▶,*b* ▶); Speier & Fulop (1989 ▶); Usubaliev *et al.* (1980 ▶).
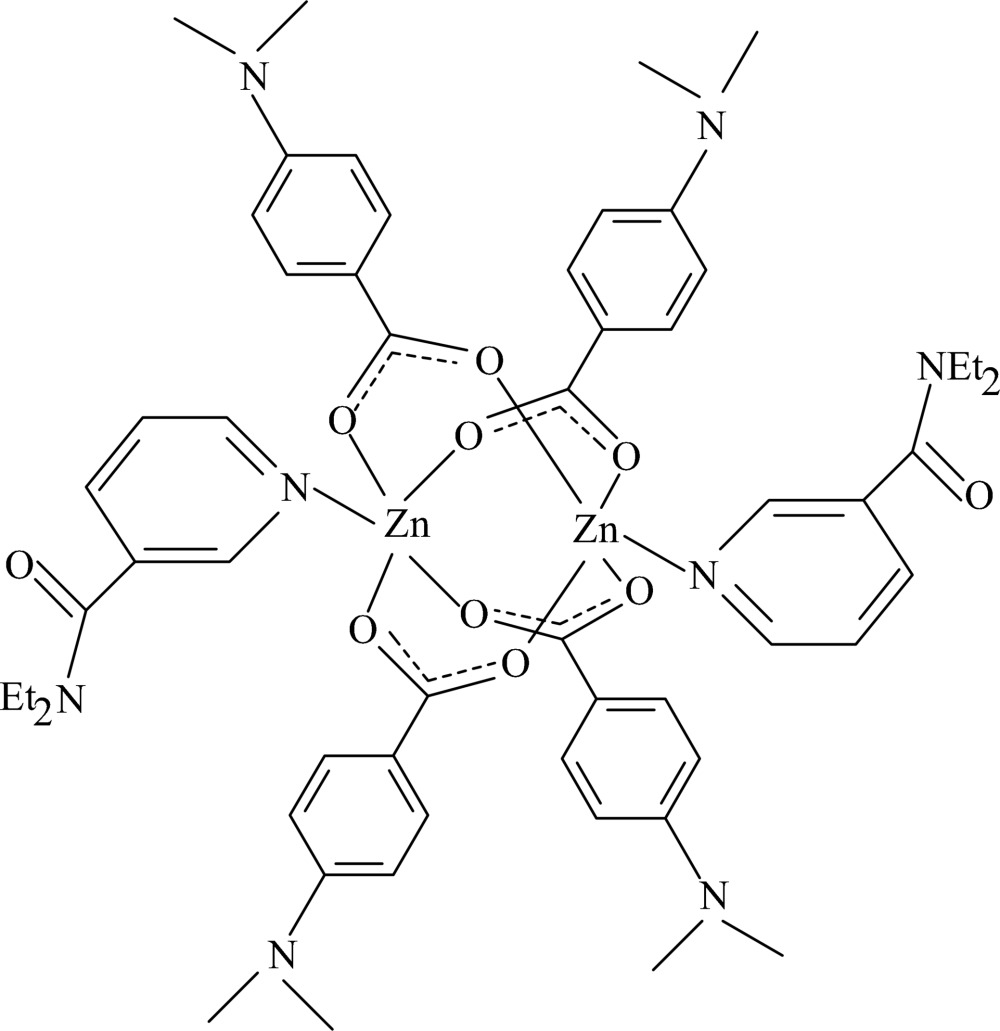


## Experimental

### 

#### Crystal data

[Zn_2_(C_9_H_10_NO_2_)_4_(C_10_H_14_N_2_O)_2_]
*M* *_r_* = 1143.96
Triclinic, 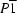

*a* = 9.2731 (6) Å
*b* = 13.2340 (8) Å
*c* = 13.4756 (8) Å
α = 112.348 (3)°
β = 109.236 (2)°
γ = 95.728 (2)°
*V* = 1395.33 (16) Å^3^
*Z* = 1
Mo *K*α radiation
μ = 0.92 mm^−1^
*T* = 294 K
0.52 × 0.35 × 0.25 mm

#### Data collection

Bruker Kappa APEXII CCD area-detector diffractometer
Absorption correction: multi-scan (*SADABS*; Bruker, 2005 ▶) *T*_min_ = 0.681, *T*_max_ = 0.791
24970 measured reflections
6877 independent reflections
5749 reflections with *I* > 2σ(*I*)
*R*_int_ = 0.029

#### Refinement

*R*[*F* ^2^ > 2σ(*F* ^2^)] = 0.033
*wR*(*F* ^2^) = 0.090
*S* = 1.06
6877 reflections
349 parameters
H-atom parameters constrained
Δρ_max_ = 0.24 e Å^−3^
Δρ_min_ = −0.43 e Å^−3^

### 

Data collection: *APEX2* (Bruker, 2007 ▶); cell refinement: *SAINT* (Bruker, 2007 ▶); data reduction: *SAINT*; program(s) used to solve structure: *SHELXS97* (Sheldrick, 2008 ▶); program(s) used to refine structure: *SHELXL97* (Sheldrick, 2008 ▶); molecular graphics: *ORTEP-3 for Windows* (Farrugia, 1997 ▶); software used to prepare material for publication: *WinGX* (Farrugia, 1999 ▶) and *PLATON* (Spek, 2009 ▶).

## Supplementary Material

Crystal structure: contains datablocks I, global. DOI: 10.1107/S1600536809047473/xu2669sup1.cif

Structure factors: contains datablocks I. DOI: 10.1107/S1600536809047473/xu2669Isup2.hkl

Additional supplementary materials:  crystallographic information; 3D view; checkCIF report

## Figures and Tables

**Table 1:** Selected bond lengths (Å)

Zn1—O1	2.0265 (12)
Zn1—O2	2.0269 (12)
Zn1—O4	2.0669 (12)
Zn1—O5	2.0459 (12)
Zn1—N3	2.0446 (13)

**Table 2:** Hydrogen-bond geometry (Å, °)

*D*—H⋯*A*	*D*—H	H⋯*A*	*D*⋯*A*	*D*—H⋯*A*
C23—H23⋯O5	0.93	2.54	3.122 (2)	121
C8—H8*A*⋯*Cg*3^i^	0.96	2.77	3.629 (3)	150

Symmetry code: (i) 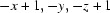. *Cg*3 is the centroid of the N3/C19–C23 ring.

**Fig. 1.**
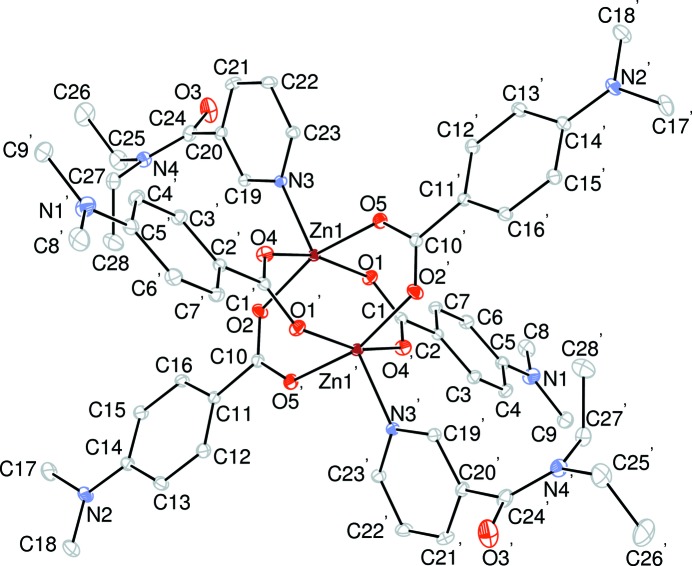
The molecular structure of the title molecule with the atom-numbering scheme. Displacement ellipsoids are drawn at the 15% probability level. Primed atoms are generated by the symmetry operator:(') 1-x, 1-y, 1-z.

## supplementary crystallographic information

### Comment

As a part of our ongoing investigation on transition metal complexes of nicotinamide (NA), one form of niacin (Krishnamachari, 1974), and/or the nicotinic acid derivative *N*,*N*-diethylnicotinamide (DENA), an important respiratory stimulant (Bigoli *et al.*, 1972), the title compound was synthesized and its crystal structure is reported herein.

The title compound is a binuclear compound, consisting of two DENA and four dimethylaminobenzoate (DMAB) ligands. The crystal structures of similar complexes of Cu^2+^ and Zn^2+^ ions, [Cu(C_6_H_5_COO)_2_(C_5_H_5_N)]_2_ (Usubaliev *et al.*, 1980); [Cu(C_6_H_5_CO_2_)_2_(py)]_2_ (Speier & Fulop, 1989), [Cu_2_(C_6_H_5_COO)_4_(C_10_H_14_N_2_O)_2_] (Hökelek *et al.*, 1995), [Zn_2_(C_11_H_14_NO_2_)_4_(C_10_H_14_N_2_O)_2_] (Hökelek *et al.*, 2009*a*) and [Zn_2_(C_8_H_8_NO_2_)_4_(C_10_H_14_N_2_O)_2_].2H_2_O (Hökelek *et al.*, 2009*b*) have also been determined. In these structures, the benzoate ion acts as a bidentate ligand.

The title dimeric complex, [Zn_2_(DMAB)_4_(DENA)_2_], has a centre of symmetry and two Zn^II^ atoms surrounded by four DMAB groups and two DENA ligands (Fig. 1). The DENA ligands are coordinated to Zn atoms through pyridine N atoms only. The DMAB groups act as bridging ligands. The Zn···Zn' distance is 2.8927 (4) Å. The average Zn—O distance is 2.0416 (12) Å (Table 1), and four O atoms of the bridging DMAB ligands around each Zn atom form a distorted square plane. The Zn atom lies 0.3326 (2) Å below the least-squares plane. The average O—Zn—O bond angle is 88.48 (6)°. A distorted square-pyramidal arrangement around each Zn atom is completed by the pyridine N atom of DENA ligand at 2.0446 (13) Å from the Zn atom. The N3—Zn1···Zn1' angle is 163.64 (6)° and the dihedral angle between plane through Zn1, O1, O4, C1, Zn1', O1', O4', C1' and the plane through Zn1, O2, O5, C10, Zn1', O2', O5', C10' is 89.47 (7)°. The dihedral angles between the planar carboxylate groups and the adjacent benzene rings A (C2—C7) and B (C11—C16) are 5.31 (8)° and 11.00 (9)°, respectively, while that between rings A and B is A/B = 83.70 (6)°. Ring C (N3/C19—C23) is oriented with respect to rings A and B at dihedral angles A/C = 66.26 (6) and B/C = 37.88 (7) °.

Weak intramolecular C—H···O and C—H···π interactions (Table 2) are present, in which they may be effective in the stabilization of the structure.

### Experimental

The title compound was prepared by the reaction of ZnSO_4_.H_2_O (0.9 g, 5 mmol) in H_2_O (50 ml) and DENA (1.78 g, 10 mmol) in H_2_O (50 ml) with sodium *p*-dimethylaminobenzoate (1.88 g, 10 mmol) in H_2_O (100 ml). The mixture was filtered and set aside to crystallize at ambient temperature for one week, giving colorless single crystals.

### Refinement

H atoms were positioned geometrically with C—H = 0.93, 0.97 and 0.96 Å, for aromatic, methylene and methyl H atoms, respectively, and constrained to ride on their parent atoms, with *U*_iso_(H) = *xU*_eq_(C), where *x* = 1.5 for methyl H and *x* = 1.2 for all other H atoms.

### Crystal data

**Table:**

[Zn_2_(C_9_H_10_NO_2_)_4_(C_10_H_14_N_2_O)_2_]	*Z* = 1
*M**_r_* = 1143.96	*F*(000) = 600
Triclinic, *P*1	*D*_x_ = 1.361 Mg m^−^^3^
Hall symbol: -P 1	Mo *K*α radiation, λ = 0.71073 Å
*a* = 9.2731 (6) Å	Cell parameters from 9870 reflections
*b* = 13.2340 (8) Å	θ = 2.4–28.3°
*c* = 13.4756 (8) Å	µ = 0.92 mm^−^^1^
α = 112.348 (3)°	*T* = 294 K
β = 109.236 (2)°	Block, colorless
γ = 95.728 (2)°	0.52 × 0.35 × 0.25 mm
*V* = 1395.33 (16) Å^3^	

### Data collection

**Table:**

Bruker Kappa APEXII CCD area-detector diffractometer	6877 independent reflections
Radiation source: fine-focus sealed tube	5749 reflections with *I* > 2σ(*I*)
graphite	*R*_int_ = 0.029
φ and ω scans	θ_max_ = 28.3°, θ_min_ = 1.7°
Absorption correction: multi-scan (*SADABS*; Bruker, 2005)	*h* = −12→12
*T*_min_ = 0.681, *T*_max_ = 0.791	*k* = −16→17
24970 measured reflections	*l* = −17→17

### Refinement

**Table:**

Refinement on *F*^2^	Primary atom site location: structure-invariant direct methods
Least-squares matrix: full	Secondary atom site location: difference Fourier map
*R*[*F*^2^ > 2σ(*F*^2^)] = 0.033	Hydrogen site location: inferred from neighbouring sites
*wR*(*F*^2^) = 0.090	H-atom parameters constrained
*S* = 1.06	*w* = 1/[σ^2^(*F*_o_^2^) + (0.0476*P*)^2^ + 0.2013*P*] where *P* = (*F*_o_^2^ + 2*F*_c_^2^)/3
6877 reflections	(Δ/σ)_max_ = 0.001
349 parameters	Δρ_max_ = 0.24 e Å^−^^3^
0 restraints	Δρ_min_ = −0.43 e Å^−^^3^

### Special details

**Table:**

Geometry. All e.s.d.'s (except the e.s.d. in the dihedral angle between two l.s. planes) are estimated using the full covariance matrix. The cell e.s.d.'s are taken into account individually in the estimation of e.s.d.'s in distances, angles and torsion angles; correlations between e.s.d.'s in cell parameters are only used when they are defined by crystal symmetry. An approximate (isotropic) treatment of cell e.s.d.'s is used for estimating e.s.d.'s involving l.s. planes.
Refinement. Refinement of *F*^2^ against ALL reflections. The weighted *R*-factor *wR* and goodness of fit *S* are based on *F*^2^, conventional *R*-factors *R* are based on *F*, with *F* set to zero for negative *F*^2^. The threshold expression of *F*^2^ > σ(*F*^2^) is used only for calculating *R*-factors(gt) *etc*. and is not relevant to the choice of reflections for refinement. *R*-factors based on *F*^2^ are statistically about twice as large as those based on *F*, and *R*- factors based on ALL data will be even larger.

### Fractional atomic coordinates and isotropic or equivalent isotropic displacement parameters (Å^2^)

**Table:**

	*x*	*y*	*z*	*U*_iso_*/*U*_eq_	
Zn1	0.647429 (19)	0.587023 (15)	0.565621 (14)	0.03318 (7)	
N1	0.0447 (2)	0.97057 (18)	0.3697 (2)	0.0740 (6)	
N2	0.3784 (2)	0.63356 (17)	1.11287 (14)	0.0626 (5)	
N3	0.88138 (15)	0.67128 (11)	0.64273 (11)	0.0336 (3)	
N4	1.16916 (19)	0.90139 (14)	1.01779 (14)	0.0529 (4)	
O1	0.52598 (14)	0.69380 (11)	0.52284 (11)	0.0495 (3)	
O2	0.58984 (16)	0.62604 (11)	0.70601 (11)	0.0514 (3)	
O3	1.2203 (3)	1.00113 (14)	0.92564 (15)	0.0984 (7)	
O4	0.69851 (15)	0.44310 (11)	0.57910 (12)	0.0515 (3)	
O5	0.63255 (15)	0.51278 (12)	0.39761 (10)	0.0542 (3)	
C1	0.3792 (2)	0.65704 (15)	0.46130 (14)	0.0387 (4)	
C2	0.29154 (19)	0.73827 (14)	0.43619 (14)	0.0375 (4)	
C3	0.1306 (2)	0.70435 (16)	0.36591 (16)	0.0466 (4)	
H3	0.0769	0.6287	0.3333	0.056*	
C4	0.0491 (2)	0.77940 (18)	0.34350 (18)	0.0533 (5)	
H4	−0.0584	0.7536	0.2961	0.064*	
C5	0.1243 (2)	0.89438 (17)	0.39039 (18)	0.0499 (4)	
C6	0.2864 (2)	0.92796 (17)	0.46138 (18)	0.0512 (5)	
H6	0.3409	1.0035	0.4947	0.061*	
C7	0.3659 (2)	0.85148 (16)	0.48263 (16)	0.0453 (4)	
H7	0.4734	0.8767	0.5299	0.054*	
C8	0.1256 (3)	1.0887 (2)	0.4196 (2)	0.0767 (7)	
H8A	0.0508	1.1301	0.3992	0.115*	
H8B	0.2045	1.0961	0.3895	0.115*	
H8C	0.1752	1.1183	0.5032	0.115*	
C9	−0.1234 (3)	0.9381 (2)	0.3032 (2)	0.0778 (7)	
H9A	−0.1586	1.0036	0.3019	0.117*	
H9B	−0.1759	0.9056	0.3388	0.117*	
H9C	−0.1478	0.8836	0.2247	0.117*	
C10	0.4677 (2)	0.56674 (16)	0.69715 (14)	0.0418 (4)	
C11	0.43798 (19)	0.58899 (15)	0.80439 (14)	0.0398 (4)	
C12	0.3197 (2)	0.51570 (17)	0.80381 (15)	0.0486 (4)	
H12	0.2529	0.4548	0.7334	0.058*	
C13	0.2979 (2)	0.53000 (18)	0.90376 (17)	0.0531 (5)	
H13	0.2169	0.4791	0.8997	0.064*	
C14	0.3962 (2)	0.62048 (17)	1.01199 (15)	0.0478 (4)	
C15	0.5102 (2)	0.69743 (18)	1.01119 (15)	0.0530 (5)	
H15	0.5735	0.7608	1.0805	0.064*	
C16	0.5308 (2)	0.68143 (17)	0.91032 (15)	0.0474 (4)	
H16	0.6087	0.7337	0.9131	0.057*	
C17	0.4929 (3)	0.7180 (2)	1.22593 (18)	0.0795 (7)	
H17A	0.4760	0.7042	1.2867	0.119*	
H17B	0.4808	0.7918	1.2350	0.119*	
H17C	0.5977	0.7140	1.2310	0.119*	
C18	0.2607 (3)	0.5536 (2)	1.1128 (2)	0.0754 (7)	
H18A	0.2701	0.5743	1.1916	0.113*	
H18B	0.2763	0.4793	1.0808	0.113*	
H18C	0.1572	0.5539	1.0661	0.113*	
C19	0.94694 (19)	0.75356 (14)	0.75189 (14)	0.0369 (4)	
H19	0.8823	0.7750	0.7921	0.044*	
C20	1.1054 (2)	0.80823 (15)	0.80792 (15)	0.0427 (4)	
C21	1.2004 (2)	0.77375 (18)	0.74711 (18)	0.0539 (5)	
H21	1.3080	0.8089	0.7816	0.065*	
C22	1.1348 (2)	0.68765 (18)	0.63601 (18)	0.0537 (5)	
H22	1.1976	0.6621	0.5951	0.064*	
C23	0.9753 (2)	0.63968 (15)	0.58606 (15)	0.0416 (4)	
H23	0.9305	0.5829	0.5097	0.050*	
C24	1.1705 (2)	0.91161 (17)	0.92357 (17)	0.0536 (5)	
C25	1.2270 (3)	1.0051 (2)	1.1274 (2)	0.0837 (8)	
H25A	1.1758	0.9954	1.1766	0.100*	
H25B	1.1973	1.0667	1.1101	0.100*	
C26	1.4002 (4)	1.0362 (3)	1.1927 (3)	0.1230 (13)	
H26A	1.4314	1.1060	1.2619	0.184*	
H26B	1.4518	1.0449	1.1442	0.184*	
H26C	1.4299	0.9777	1.2144	0.184*	
C27	1.1366 (3)	0.79420 (19)	1.02381 (18)	0.0609 (5)	
H27A	1.2212	0.7969	1.0912	0.073*	
H27B	1.1368	0.7341	0.9545	0.073*	
C28	0.9820 (4)	0.7661 (3)	1.0327 (3)	0.0990 (9)	
H28A	0.9775	0.7036	1.0522	0.149*	
H28B	0.8967	0.7463	0.9591	0.149*	
H28C	0.9729	0.8306	1.0926	0.149*	

### Atomic displacement parameters (Å^2^)

**Table:**

	*U*^11^	*U*^22^	*U*^33^	*U*^12^	*U*^13^	*U*^23^
Zn1	0.02904 (10)	0.03429 (12)	0.03032 (10)	0.00543 (7)	0.01153 (7)	0.00929 (8)
N1	0.0647 (12)	0.0723 (13)	0.1079 (16)	0.0331 (10)	0.0319 (11)	0.0608 (12)
N2	0.0671 (11)	0.0820 (13)	0.0420 (9)	0.0147 (10)	0.0313 (8)	0.0238 (9)
N3	0.0325 (6)	0.0324 (7)	0.0342 (7)	0.0087 (5)	0.0142 (5)	0.0120 (6)
N4	0.0554 (10)	0.0441 (9)	0.0426 (8)	0.0082 (7)	0.0161 (7)	0.0065 (7)
O1	0.0403 (7)	0.0488 (8)	0.0577 (8)	0.0151 (6)	0.0144 (6)	0.0253 (6)
O2	0.0586 (8)	0.0561 (8)	0.0402 (7)	0.0107 (6)	0.0297 (6)	0.0145 (6)
O3	0.1420 (17)	0.0466 (10)	0.0616 (10)	−0.0197 (10)	0.0089 (10)	0.0166 (8)
O4	0.0514 (7)	0.0395 (7)	0.0664 (8)	0.0141 (6)	0.0237 (6)	0.0253 (6)
O5	0.0479 (7)	0.0726 (9)	0.0298 (6)	0.0141 (7)	0.0151 (5)	0.0108 (6)
C1	0.0444 (9)	0.0414 (10)	0.0387 (9)	0.0150 (7)	0.0233 (7)	0.0191 (8)
C2	0.0395 (8)	0.0417 (9)	0.0381 (8)	0.0118 (7)	0.0197 (7)	0.0202 (7)
C3	0.0426 (9)	0.0424 (10)	0.0513 (10)	0.0073 (8)	0.0159 (8)	0.0204 (8)
C4	0.0403 (9)	0.0593 (13)	0.0585 (12)	0.0129 (9)	0.0130 (8)	0.0296 (10)
C5	0.0522 (11)	0.0547 (12)	0.0596 (12)	0.0221 (9)	0.0271 (9)	0.0358 (10)
C6	0.0538 (11)	0.0411 (10)	0.0640 (12)	0.0108 (8)	0.0240 (9)	0.0284 (9)
C7	0.0398 (9)	0.0465 (10)	0.0510 (10)	0.0085 (8)	0.0173 (8)	0.0241 (9)
C8	0.0995 (19)	0.0644 (16)	0.0989 (19)	0.0435 (14)	0.0491 (16)	0.0549 (15)
C9	0.0682 (15)	0.104 (2)	0.0950 (19)	0.0481 (15)	0.0366 (14)	0.0672 (17)
C10	0.0423 (9)	0.0517 (11)	0.0345 (8)	0.0217 (8)	0.0190 (7)	0.0166 (8)
C11	0.0367 (8)	0.0509 (10)	0.0323 (8)	0.0161 (7)	0.0164 (7)	0.0153 (7)
C12	0.0471 (10)	0.0517 (11)	0.0359 (9)	0.0081 (8)	0.0150 (8)	0.0107 (8)
C13	0.0522 (11)	0.0602 (12)	0.0469 (10)	0.0069 (9)	0.0250 (9)	0.0210 (9)
C14	0.0488 (10)	0.0608 (12)	0.0391 (9)	0.0194 (9)	0.0243 (8)	0.0203 (9)
C15	0.0483 (10)	0.0634 (13)	0.0322 (9)	0.0059 (9)	0.0166 (8)	0.0075 (9)
C16	0.0410 (9)	0.0574 (12)	0.0374 (9)	0.0070 (8)	0.0191 (7)	0.0128 (8)
C17	0.0939 (18)	0.100 (2)	0.0390 (11)	0.0174 (15)	0.0317 (12)	0.0223 (12)
C18	0.0869 (17)	0.0942 (19)	0.0689 (15)	0.0242 (14)	0.0474 (14)	0.0456 (15)
C19	0.0358 (8)	0.0342 (9)	0.0345 (8)	0.0068 (7)	0.0140 (6)	0.0097 (7)
C20	0.0384 (9)	0.0404 (10)	0.0390 (9)	0.0039 (7)	0.0071 (7)	0.0160 (8)
C21	0.0300 (8)	0.0586 (12)	0.0637 (12)	0.0058 (8)	0.0136 (8)	0.0234 (10)
C22	0.0401 (9)	0.0651 (13)	0.0621 (12)	0.0180 (9)	0.0298 (9)	0.0249 (10)
C23	0.0402 (9)	0.0431 (10)	0.0391 (9)	0.0123 (7)	0.0197 (7)	0.0122 (8)
C24	0.0504 (11)	0.0419 (11)	0.0454 (10)	0.0019 (8)	0.0044 (8)	0.0113 (8)
C25	0.108 (2)	0.0578 (15)	0.0513 (13)	0.0038 (14)	0.0278 (13)	−0.0016 (11)
C26	0.110 (3)	0.107 (3)	0.0625 (17)	−0.029 (2)	−0.0102 (16)	0.0001 (16)
C27	0.0666 (13)	0.0592 (13)	0.0478 (11)	0.0142 (10)	0.0150 (10)	0.0215 (10)
C28	0.100 (2)	0.110 (2)	0.110 (2)	0.0161 (18)	0.0574 (19)	0.061 (2)

### Geometric parameters (Å, °)

**Table:**

Zn1—Zn1^i^	2.8927 (4)	C11—C16	1.388 (2)
Zn1—O1	2.0265 (12)	C12—C13	1.373 (3)
Zn1—O2	2.0269 (12)	C12—H12	0.9300
Zn1—O4	2.0669 (12)	C13—H13	0.9300
Zn1—O5	2.0459 (12)	C14—N2	1.371 (2)
Zn1—N3	2.0446 (13)	C14—C13	1.404 (3)
O1—C1	1.263 (2)	C14—C15	1.399 (3)
O2—C10	1.255 (2)	C15—C16	1.374 (2)
O4—C1^i^	1.255 (2)	C15—H15	0.9300
O5—C10^i^	1.266 (2)	C16—H16	0.9300
N1—C9	1.438 (3)	C17—H17A	0.9600
N1—C8	1.447 (3)	C17—H17B	0.9600
N2—C18	1.443 (3)	C17—H17C	0.9600
N2—C17	1.449 (3)	C18—H18A	0.9600
N3—C19	1.334 (2)	C18—H18B	0.9600
N3—C23	1.336 (2)	C18—H18C	0.9600
N4—C25	1.463 (3)	C19—C20	1.377 (2)
N4—C27	1.459 (3)	C19—H19	0.9300
C1—O4^i^	1.255 (2)	C20—C21	1.388 (3)
C2—C1	1.484 (2)	C20—C24	1.503 (2)
C2—C3	1.392 (2)	C21—H21	0.9300
C2—C7	1.381 (2)	C22—C21	1.369 (3)
C3—C4	1.369 (3)	C22—H22	0.9300
C3—H3	0.9300	C23—C22	1.370 (2)
C4—C5	1.403 (3)	C23—H23	0.9300
C4—H4	0.9300	C24—O3	1.214 (3)
C5—N1	1.366 (2)	C24—N4	1.331 (3)
C6—C5	1.402 (3)	C25—C26	1.478 (4)
C6—H6	0.9300	C25—H25A	0.9700
C7—C6	1.371 (3)	C25—H25B	0.9700
C7—H7	0.9300	C26—H26A	0.9600
C8—H8A	0.9600	C26—H26B	0.9600
C8—H8B	0.9600	C26—H26C	0.9600
C8—H8C	0.9600	C27—C28	1.500 (3)
C9—H9A	0.9600	C27—H27A	0.9700
C9—H9B	0.9600	C27—H27B	0.9700
C9—H9C	0.9600	C28—H28A	0.9600
C10—O5^i^	1.266 (2)	C28—H28B	0.9600
C11—C10	1.485 (2)	C28—H28C	0.9600
C11—C12	1.386 (3)		
			
O1—Zn1—O2	89.01 (6)	C13—C12—H12	118.9
O1—Zn1—O4	161.34 (5)	C12—C13—C14	120.84 (18)
O1—Zn1—O5	88.39 (6)	C12—C13—H13	119.6
O1—Zn1—N3	106.31 (5)	C14—C13—H13	119.6
O2—Zn1—O4	89.28 (6)	N2—C14—C13	121.29 (18)
O2—Zn1—O5	161.09 (6)	N2—C14—C15	121.92 (18)
O2—Zn1—N3	101.76 (5)	C15—C14—C13	116.77 (16)
O5—Zn1—O4	87.23 (6)	C16—C15—C14	121.46 (18)
N3—Zn1—O4	92.23 (5)	C16—C15—H15	119.3
N3—Zn1—O5	96.95 (5)	C14—C15—H15	119.3
C1—O1—Zn1	118.82 (11)	C11—C16—H16	119.2
C10—O2—Zn1	119.77 (11)	C15—C16—C11	121.52 (18)
C1^i^—O4—Zn1	135.03 (12)	C15—C16—H16	119.2
C10^i^—O5—Zn1	134.43 (12)	N2—C17—H17A	109.5
C5—N1—C8	120.9 (2)	N2—C17—H17B	109.5
C5—N1—C9	121.6 (2)	N2—C17—H17C	109.5
C9—N1—C8	117.41 (19)	H17A—C17—H17B	109.5
C14—N2—C17	120.85 (19)	H17A—C17—H17C	109.5
C14—N2—C18	121.51 (19)	H17B—C17—H17C	109.5
C18—N2—C17	116.96 (18)	N2—C18—H18A	109.5
C19—N3—Zn1	121.99 (11)	N2—C18—H18B	109.5
C19—N3—C23	118.04 (14)	N2—C18—H18C	109.5
C23—N3—Zn1	119.85 (11)	H18A—C18—H18B	109.5
C24—N4—C25	117.08 (19)	H18A—C18—H18C	109.5
C24—N4—C27	124.81 (17)	H18B—C18—H18C	109.5
C27—N4—C25	117.48 (18)	N3—C19—C20	123.28 (15)
O1—C1—C2	117.78 (15)	N3—C19—H19	118.4
O4^i^—C1—O1	124.74 (16)	C20—C19—H19	118.4
O4^i^—C1—C2	117.47 (15)	C19—C20—C21	117.52 (16)
C3—C2—C1	121.68 (16)	C19—C20—C24	121.55 (16)
C7—C2—C1	121.40 (15)	C21—C20—C24	120.41 (16)
C7—C2—C3	116.92 (16)	C20—C21—H21	120.2
C2—C3—H3	119.1	C22—C21—C20	119.63 (16)
C4—C3—C2	121.76 (17)	C22—C21—H21	120.2
C4—C3—H3	119.1	C21—C22—C23	118.90 (17)
C3—C4—C5	121.46 (18)	C21—C22—H22	120.6
C3—C4—H4	119.3	C23—C22—H22	120.6
C5—C4—H4	119.3	N3—C23—C22	122.60 (16)
N1—C5—C4	122.23 (19)	N3—C23—H23	118.7
N1—C5—C6	121.32 (19)	C22—C23—H23	118.7
C6—C5—C4	116.45 (17)	O3—C24—N4	123.26 (19)
C5—C6—H6	119.4	O3—C24—C20	117.73 (19)
C7—C6—C5	121.22 (18)	N4—C24—C20	119.00 (18)
C7—C6—H6	119.4	N4—C25—C26	113.3 (2)
C2—C7—H7	118.9	N4—C25—H25A	108.9
C6—C7—C2	122.19 (17)	N4—C25—H25B	108.9
C6—C7—H7	118.9	C26—C25—H25A	108.9
N1—C8—H8A	109.5	C26—C25—H25B	108.9
N1—C8—H8B	109.5	H25A—C25—H25B	107.7
N1—C8—H8C	109.5	C25—C26—H26A	109.5
H8A—C8—H8B	109.5	C25—C26—H26B	109.5
H8A—C8—H8C	109.5	C25—C26—H26C	109.5
H8B—C8—H8C	109.5	H26A—C26—H26B	109.5
N1—C9—H9A	109.5	H26A—C26—H26C	109.5
N1—C9—H9B	109.5	H26B—C26—H26C	109.5
N1—C9—H9C	109.5	N4—C27—C28	113.8 (2)
H9A—C9—H9B	109.5	N4—C27—H27A	108.8
H9A—C9—H9C	109.5	N4—C27—H27B	108.8
H9B—C9—H9C	109.5	C28—C27—H27A	108.8
O2—C10—O5^i^	124.39 (16)	C28—C27—H27B	108.8
O2—C10—C11	118.63 (15)	H27A—C27—H27B	107.7
O5^i^—C10—C11	116.98 (16)	C27—C28—H28A	109.5
C12—C11—C10	121.20 (16)	C27—C28—H28B	109.5
C12—C11—C16	117.10 (16)	C27—C28—H28C	109.5
C16—C11—C10	121.66 (16)	H28A—C28—H28B	109.5
C11—C12—H12	118.9	H28A—C28—H28C	109.5
C13—C12—C11	122.17 (17)	H28B—C28—H28C	109.5
			
O2—Zn1—O1—C1	−86.30 (13)	C3—C2—C7—C6	0.1 (3)
O4—Zn1—O1—C1	−1.5 (2)	C2—C3—C4—C5	0.0 (3)
O5—Zn1—O1—C1	74.97 (13)	C3—C4—C5—N1	−179.4 (2)
N3—Zn1—O1—C1	171.73 (12)	C3—C4—C5—C6	−0.2 (3)
O1—Zn1—O2—C10	92.13 (14)	C4—C5—N1—C8	179.9 (2)
O4—Zn1—O2—C10	−69.29 (14)	C4—C5—N1—C9	3.2 (3)
O5—Zn1—O2—C10	10.0 (3)	C6—C5—N1—C8	0.7 (3)
N3—Zn1—O2—C10	−161.42 (14)	C6—C5—N1—C9	−176.0 (2)
O1—Zn1—O4—C1^i^	4.3 (3)	C7—C6—C5—N1	179.6 (2)
O2—Zn1—O4—C1^i^	89.10 (17)	C7—C6—C5—C4	0.3 (3)
O5—Zn1—O4—C1^i^	−72.31 (17)	C2—C7—C6—C5	−0.2 (3)
N3—Zn1—O4—C1^i^	−169.16 (17)	C12—C11—C10—O2	−169.73 (18)
O1—Zn1—O5—C10^i^	−88.02 (18)	C12—C11—C10—O5^i^	9.4 (3)
O2—Zn1—O5—C10^i^	−5.8 (3)	C16—C11—C10—O2	7.8 (3)
O4—Zn1—O5—C10^i^	73.84 (18)	C16—C11—C10—O5^i^	−173.06 (17)
N3—Zn1—O5—C10^i^	165.75 (17)	C10—C11—C12—C13	175.00 (17)
O1—Zn1—N3—C19	75.41 (13)	C16—C11—C12—C13	−2.7 (3)
O1—Zn1—N3—C23	−108.69 (13)	C10—C11—C16—C15	−175.32 (17)
O2—Zn1—N3—C19	−17.02 (14)	C12—C11—C16—C15	2.3 (3)
O2—Zn1—N3—C23	158.88 (13)	C11—C12—C13—C14	−0.2 (3)
O4—Zn1—N3—C19	−106.75 (13)	C13—C14—N2—C17	171.8 (2)
O4—Zn1—N3—C23	69.15 (13)	C13—C14—N2—C18	1.6 (3)
O5—Zn1—N3—C19	165.77 (13)	C15—C14—N2—C17	−9.6 (3)
O5—Zn1—N3—C23	−18.33 (14)	C15—C14—N2—C18	−179.8 (2)
Zn1—O1—C1—O4^i^	−2.1 (2)	N2—C14—C13—C12	−178.00 (19)
Zn1—O1—C1—C2	176.98 (10)	C15—C14—C13—C12	3.3 (3)
Zn1—O2—C10—O5^i^	−5.9 (3)	N2—C14—C15—C16	177.68 (19)
Zn1—O2—C10—C11	173.11 (11)	C13—C14—C15—C16	−3.7 (3)
Zn1—N3—C19—C20	176.95 (13)	C14—C15—C16—C11	0.9 (3)
Zn1—N3—C23—C22	−175.48 (15)	N3—C19—C20—C21	−1.0 (3)
C19—N3—C23—C22	0.6 (3)	N3—C19—C20—C24	170.73 (17)
C23—N3—C19—C20	1.0 (2)	C19—C20—C21—C22	−0.5 (3)
C24—N4—C25—C26	−86.0 (3)	C24—C20—C21—C22	−172.34 (19)
C27—N4—C25—C26	85.3 (3)	C19—C20—C24—O3	−111.7 (2)
C24—N4—C27—C28	−110.6 (2)	C19—C20—C24—N4	67.5 (2)
C25—N4—C27—C28	78.8 (3)	C21—C20—C24—O3	59.8 (3)
C3—C2—C1—O1	177.84 (16)	C21—C20—C24—N4	−121.0 (2)
C3—C2—C1—O4^i^	−3.0 (2)	C23—C22—C21—C20	2.0 (3)
C7—C2—C1—O1	−2.8 (2)	N3—C23—C22—C21	−2.0 (3)
C7—C2—C1—O4^i^	176.30 (16)	O3—C24—N4—C25	1.9 (3)
C1—C2—C3—C4	179.40 (17)	O3—C24—N4—C27	−168.7 (2)
C7—C2—C3—C4	0.1 (3)	C20—C24—N4—C25	−177.29 (19)
C1—C2—C7—C6	−179.29 (17)	C20—C24—N4—C27	12.1 (3)

Symmetry codes: (i) −*x*+1, −*y*+1, −*z*+1.

### Hydrogen-bond geometry (Å, °)

**Table:**

*D*—H···*A*	*D*—H	H···*A*	*D*···*A*	*D*—H···*A*
C23—H23···O5	0.93	2.54	3.122 (2)	121
C8—H8A···Cg3^ii^	0.96	2.77	3.629 (3)	150

Symmetry codes: (ii) −*x*+1, −*y*, −*z*+1.
